# Association Between SGLT2 Inhibitor Use and Reduced Risk of Liver-Related Events, Including Hepatocellular Carcinoma, in Diabetic Patients with Viral Hepatitis: A Nationwide Cohort Study

**DOI:** 10.3390/cancers18010120

**Published:** 2025-12-30

**Authors:** Seong Hee Kang, Jimi Choi, Hyung Joon Yim, Young Kul Jung, Sun Young Yim, Young-Sun Lee, Yeon Seok Seo, Ji Hoon Kim, Jong Eun Yeon, Kwan Soo Byun

**Affiliations:** Department of Internal Medicine, Korea University College of Medicine, Seoul 02841, Republic of Korea

**Keywords:** hepatitis B, chronic, hepatitis C, chronic, hepatocellular carcinoma, sodium-glucose transporter 2 inhibitors, diabetes mellitus, Type 2

## Abstract

**Background and Aims**: Diabetes mellitus and chronic hepatitis B/C infection are risk factors for liver cirrhosis and hepatocellular carcinoma (HCC). This study aimed to evaluate whether sodium-glucose cotransporter-2 inhibitors (SGLT2i) improved liver-related outcomes in patients with chronic viral hepatitis and co-existing diabetes. **Methods**: Using data from the Korean nationwide cohort, this study included 37,629 patients with concurrent diabetes and chronic hepatitis B/C infection, without prior HCC, who were treated with oral hypoglycemic agents. Patients who were treated with SGLT2is for over 90 days were allocated to the SGLT2i group, whereas those who never received SGLT2is comprised the non-SGLT2i group. The primary outcome was the occurrence of liver-related events, including HCC. **Results**: After 1:2 propensity score matching, the SGLT2i group comprised 12,543 patients (chronic hepatitis B, CHB: *n* = 9392; chronic hepatitis C, CHC: *n* = 4300, CHB & CHC: *n* = 1149), while the non-SGLT2i group included 25,086 patients (CHB: *n* = 18,806; CHC: *n* = 8553, CHB & CHC: 2273). The incidence rate of composite liver-related complications was lower in the SGLT2i group than that in the non-SGLT2i group (6.67 per 1000 vs. 8.99 per 1000). Moreover, SGLT2i therapy was associated with a reduced risk of HCC development (subdistribution hazard ratio [sHR] = 0.77, 95% confidence interval [CI] = 0.66–0.91; *p* = 0.002) and developing cirrhosis (sHR = 0.67, 95% CI = 0.54–0.83; *p* < 0.001). **Conclusions**: SGLT2is should be considered a therapeutic option for controlling diabetes, reducing the metabolic burden, and improving liver outcomes in patients with concurrent diabetes and chronic viral hepatitis.

## Simple Summary

This study demonstrates that Sodium-glucose cotransporter-2 inhibitors (SGLT2i) reduce the incidence of liver-related events, including hepatocellular carcinoma (HCC), in 28,426 patients with concurrent diabetes and chronic hepatitis B/C using data from the Korean nationwide cohort. The findings suggest that SGLT2 inhibitors may play a pivotal role in reducing the burden of HCC in high-risk populations with metabolic and viral liver diseases.

## 1. Introduction

Chronic hepatitis B (CHB) and hepatitis C (CHC) infections are leading causes of liver-related morbidities including HCC [1,2]. Metabolic disorders, including Type 2 diabetes mellitus (T2DM) and obesity, play a prominent role in the disease course of CHB and CHC [3,4,5]. Moreover, recently, the landscape of HCC risk factors has shifted, with T2DM and metabolic dysfunction-associated steatotic liver disease (MASLD) emerging as significant contributors [6,7]. These conditions are increasingly recognized as major pathogenic promoters of HCC, potentially overtaking traditional causes such as viral hepatitis and alcohol-related liver disease. This shift highlights the importance of understanding and managing metabolic disorders to prevent liver cirrhosis and HCC [8].

Sodium-glucose cotransporter-2 inhibitors (SGLT2is) are a class of oral medications primarily used to manage hyperglycemia in individuals with T2DM. These drugs block glucose reabsorption in the proximal renal tubules, thus promoting glycosuria and lowering blood glucose levels [9]. Beyond maintaining glycemic control, SGLT2is are beneficial in reducing renal and cardiovascular risks, relevant for patients with T2DM [10,11].

Recent studies suggest that SGLT2is have protective effects against various liver diseases. Nationwide cohort studies have shown an association between the use of SGLT2is and MASLD regression, as well as a reduced incidence of adverse liver-related outcomes [12]. These findings are supported by Mendelian randomization analyses conducted in European and Korean cohorts, which indicate that SGLT2is might reduce the risk of liver-related complications, such as HCC [13]. Furthermore, several clinical studies have demonstrated that SGLT2is alleviate hepatic steatosis and also reduce liver inflammation and fibrosis [14,15].

Given the increasing prevalence of T2DM and MASLD, exploring the preventive effects of SGLT2is on chronic viral hepatitis is a promising research area. Therefore, this nationwide cohort study aimed to investigate the association between SGLT2is and the incidence of liver-related events, including HCC, in an exclusive cohort of patients with CHB/CHC and co-existing T2DM.

## 2. Materials and Methods

### 2.1. Study Cohort

This study used the nationwide claims data from the Korean National Health Insurance Service (NHIS)-National Health Information Database, a compulsory medical insurance system covering all citizens of South Korea. This database contains longitudinal information, including patient demographics, as well as medical and pharmaceutical records, such as the disease codes according to the International Classification of Disease, Tenth Revision (ICD-10), medical procedures, hospitalization, prescribed drugs, health screening results, and death records.

The inclusion criteria of the cohort were as follows: (1) patients aged >20 years; (2) a T2DM diagnosis (ICD-10 codes, E11–14) along with treatment with at least one oral glucose-lowering agent from 1 September 2014 (the date on which SGLT2is were first released in Korea) to 31 December 2021; and (3) a CHB (ICD-10 codes, B18.0 and B18.10) or CHC (ICD-10 codes B18.2) diagnosis. The oral antidiabetic drugs (OADs) included in this study were SGLT2is, metformin, sulfonylurea, thiazolidinedione (TZD), and dipeptidyl peptidase-4 inhibitors (DPP4is). Additionally, we included patients who had previously received insulin formulations. Overall, this study cohort comprised 131,896 patients diagnosed with T2DM and CHB or CHC infection. Individuals with a previous history of HCC or end-stage renal disease, those who underwent a liver transplantation, and those who initiated SGLT2is before the diagnosis of CHB or CHC, were excluded.

**Figure 1:**
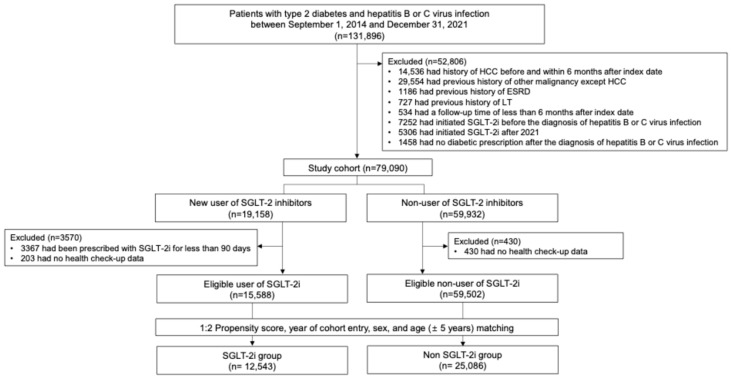
Patient flow diagram.

Based on the use of SGLT2is, participants were divided into the SGLT2i and non-SGLT2i groups. The index date of the SGLT2i group was defined as the date when the SGLT2is were first prescribed. We identified patients taking SGLT2i who were prescribed the drugs more than 90 days prior to the index date. To avoid immortal time bias, all SGLT2i non-users who were alive at the time of the index date of the SGLT2i group were selected using a risk-set sampling method with replacement. The index date for each non-user was aligned to that of the matched SGLT2i user, ensuring synchronized cohort entry and follow-up initiation across groups. We matched treated patients (SGLT2i group) with untreated patients (non-SGLT2i group) based on age, sex, baseline comorbidities, and medication history. Untreated patients were defined as never having been users of SGLT2 inhibitors, with clinical characteristics comparable to those of treated patients at the time of matching. The index date of the selected non-SGLT2i patient group was defined as July 1 of the index year of the SGLT2i group. The time from cohort entry to index date was well balanced between groups (median [IQR]: 1.9 [0.5–4.8] years in non-users vs. 2.0 [0.3–4.9] years in users; absolute standardized difference = 0.009). Participants who died or developed HCC within 6 months after the index date were excluded from the study. Propensity scores (PSs) were calculated for both the SGLT2i and non-SGLT2i groups at their respective index dates. Patients in the SGLT2i group were matched with one or two patients in the non-SGLT2i group using PS matching. The disposition of the patients in this study is shown in Figure 1.

This study was approved by the Institutional Review Board for Human Research of the Korea University Ansan Hospital (2024AS0135), and the study protocol adhered to the principles of the Declaration of Helsinki. The need for informed consent was waived because the NHIS database was anonymized and maintained with strict confidentiality. All authors had access to the study data and have reviewed and approved the final manuscript.

### 2.2. Outcome Measures

The primary outcome of this study was the development of composite liver-related compositions, including the occurrence of HCC (ICD-10 code C22.0) or cirrhosis-related complications that required admission (i.e., ascites, variceal bleeding, hepatic encephalopathy, spontaneous bacterial peritonitis, and hepatorenal syndrome), liver transplantation, or liver-related mortality. The secondary outcomes were all-cause mortality and the development of cirrhosis in patients without cirrhosis. Individuals were followed up until the cutoff date (31 December 2022), achievement of the primary outcome, or confirmation of death (Supplementary Figure S1). Cirrhosis was defined by the ICD-10 cirrhosis diagnostic code or the development of cirrhosis-related complications.

### 2.3. Confounding Variables

Confounding variables in this study included patient age at the index date; sex; socioeconomic status (SES); duration of diabetes or chronic hepatitis; comorbidities such as cancer, liver cirrhosis, hypertension, dyslipidemia, and cardiovascular disease; and the Charlson’s comorbidity index. Prescribed drugs, including antidiabetics, antihypertensives, statins, other lipid-lowering agents, and antithrombotic agents, were defined as those used for >30 days before the index year. Comorbidities and concurrent drug treatment were defined based on the ICD codes or medication use identified within 1 year prior to the index date. Diabetes duration (in years) was calculated from the date of the first medical treatment based on a diabetes diagnosis to the event index date.

### 2.4. Statistical Analyses

Continuous variables are presented as means ± standard deviations or medians (interquartile ranges [IQRs]), and categorical variables are described as numbers with percentages. The PS matching at a 1:1–2 matching was used to reduce the effects of confounders between the SGLT2i and non-SGLT2i groups. We used the absolute standardized mean difference (ASMD) to evaluate the balance between the two groups for baseline covariates. An ASMD threshold <0.1 was used to indicate adequate balance, ensuring that the confounding variables were minimized. The PSs were derived from a multiple logistic regression model that included all variables listed in the baseline characteristics (Table 1): age, sex, time to index date from entry into the cohort, diagnosis of diabetes or chronic hepatitis, SES, comorbidities, concurrent drug treatment, and the presence of metabolic dysfunction-associated steatotic liver disease (MASLD) as defined by a Fatty Liver Index (FLI) ≥ 60. We used greedy nearest-neighbor matching on the logit of the PS with a caliper width of 0.2 times the standard deviation of the logit. The incidence rates of the study outcomes were calculated as the number of events per 1000 person-years. To account for competing risks from non–liver-related death or liver transplantation, the cumulative incidence of liver-related outcomes was graphically presented using a cumulative incidence function (CIF) curve. The relative subdistribution hazard of events in the SGLT2i group compared with the non-SGLT2i group was estimated to use the Fine and Gray subdistribution hazards model with a robust sandwich variance estimator to account for intra-cluster correlation in the matched sample. The results were expressed as subdistribution hazard ratios (SHR) and 95% confidence intervals (CI). A two-sided *p*-value < 0.05 was considered statistically significant. All statistical analyses were performed using SAS software version 9.4, SAS Enterprise Guide software, version 7.1 (SAS Institute Inc., Cary, NC, USA).

## 3. Results

### 3.1. Subsection

Based on the study criteria, we included 19,158 eligible SGLT2i users and 59,932 non-users by risk-set sampling at the time of the index date of the SGLT2i users. Detailed information on all participants before PS matching is presented in Supplementary Table S1. After PS matching, the baseline characteristics between the SGLT2i group (*n* = 12,543) and the non-SGLT2i group (*n* = 25,086) as well as all variables, including the distribution of health check-up dates, showed an ASMD of <0.1, indicating well-balanced differences (Table 1). The SGLT2i and non-SGLT2i groups had 9392 (74.9%) and 18,806 (75.0%) patients with CHB, and 4300 (34.3%) and 8553 (34.1%) patients with CHC, respectively. Also, patients with concurrent CHB and CHC were included: 9.2% in the SGLT2i group (*n* = 1149) and 9.1% in the non-SGLT2i group (*n* = 2273). Hepatic steatosis and liver cirrhosis at baseline were present in 39.7% and 5.6% of the SGLT2 inhibitor group and 37.2% and 6.2% of the non-SGLT2 inhibitor group, respectively.

The frequencies of concurrent drug treatment with metformin, DPP4is, sulfonylurea, TZD, and glucagon-like peptide-1 agonists were 84.0%, 56.5%, 38.0%, 9.7%, and 0.7% in the SGLT2i group and 82.7%, 54.1%, 37.1%, 9.3%, and 0.4% in the non-SGLT2i group, respectively.

### 3.2. Risk of Composite Liver-Related Complications Including HCC

**Figure 2:**
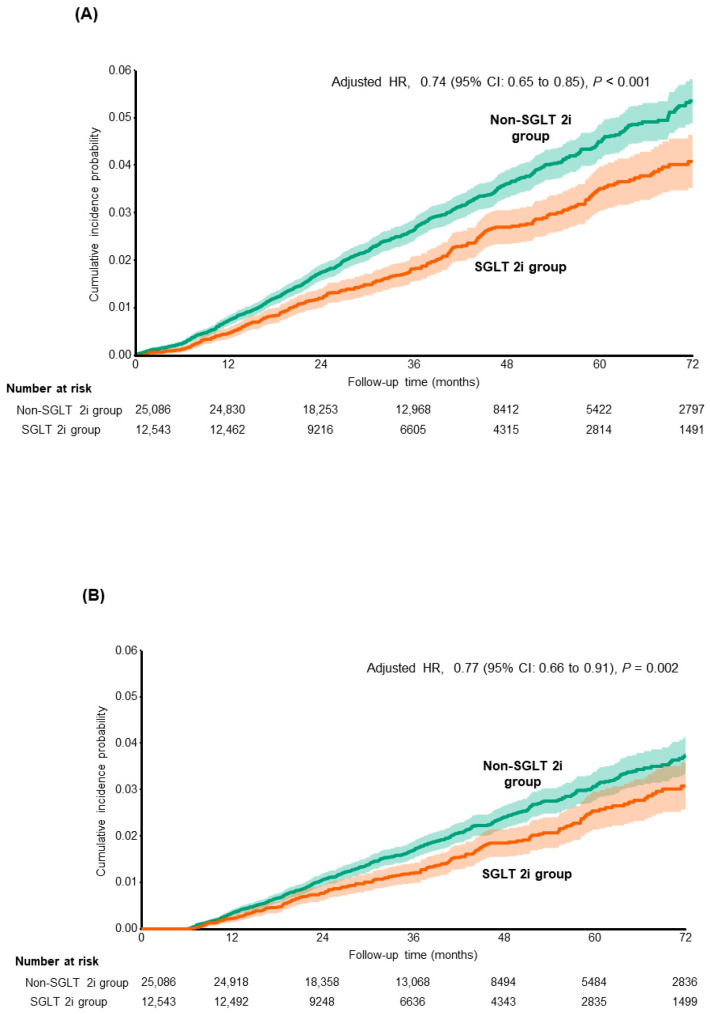

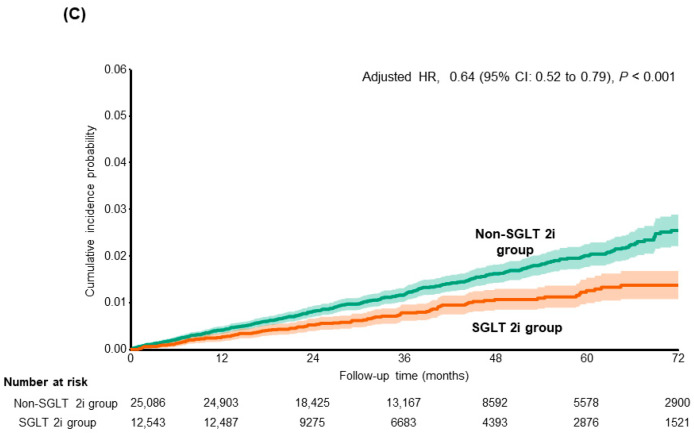
Cumulative incidence function (CIF) curves for the study outcomes after propensity score matching. The CIF represents the estimated probability of each outcome occurring over time, accounting for death as a competing risk. The hazard ratios (HRs) are provided as a measure of the relative risk between the SGLT2i user group and the non-user group. The solid lines represent the cumulative incidence for each group, and the shaded areas indicate the 95% confidence intervals (CIs). (**A**) Composite liver-related complications; (**B**) Hepatocellular carcinoma; (**C**) Cirrhosis-related complications.

The study population was followed up for a median of 3.5 (IQR, 1.9–4.5) years and 132,352 person-years. During the follow-up period, 1063 (2.8%) patients experienced composite liver-related complications, including HCC. The incidence was lower in the SGLT2i group than in the non-SGLT2i group (sHR = 0.74, 95% CI = 0.65–0.85; *p* < 0.001) (Figure 2A), with incidence rates of 6.67 per 1000 person-years and 8.99 per 1000 person-years, respectively (Table 2). For further analysis, we separated overall liver-related complications into liver-related mortality, transplantation, HCC, and cirrhosis-related complications and showed similar results. Across the study population, 717 patients developed HCC, and 473 patients presented cirrhosis-related complications. SGLT2i therapy showed a robust association with a significantly reduced risk of HCC (sHR = 0.77, 95% CI = 0.66–0.91; *p* = 0.002) (Figure 2B). Moreover, there is a significant difference in the risk of cirrhosis-related complications that were observed between the SGLT2i and the non-SGLT2i groups (sHR = 0.64, 95% CI = 0.52–0.79; *p* = 0.08) (Figure 2C). Treatment with SGLT2 inhibitors was significantly associated with a lower risk of transplantation (sHR = 0.44, 95% CI = 0.24–0.81; *p* = 0.008) as well as liver-related mortality (sHR = 0.67, 95% CI = 0.50–0.91; *p* = 0.010).

We employed a negative control outcome to increase confidence in the effect of SGLT2is on HCC development. No significant association was observed between SGLT2i treatment and the risk of other types of cancer in this cohort, including cholangiocarcinoma, pancreatic cancer, gallbladder cancer, colon cancer, gastric cancer, ampullary cancer, and bile duct cancer (Supplementary Table S2). This result supports the absence of significant residual bias from unmeasured confounding factors in the comparison.

### 3.3. Risk of Mortality and Development of Cirrhosis

Across the study population, 35,363 individuals did not have liver cirrhosis at baseline. Among these patients, those included in the SGLT2i group had a lower likelihood of developing cirrhosis than those in the non-SGLT2i group (sHR = 0.67, 95% CI = 0.54–0.83; *p* < 0.001). The SGLT2i group tends to be associated with a reduced risk of overall mortality (HR = 0.76, 95% CI = 0.65–0.88; *p* < 0.001) (Table 3).

### 3.4. Subgroup Analyses

**Figure 3:**
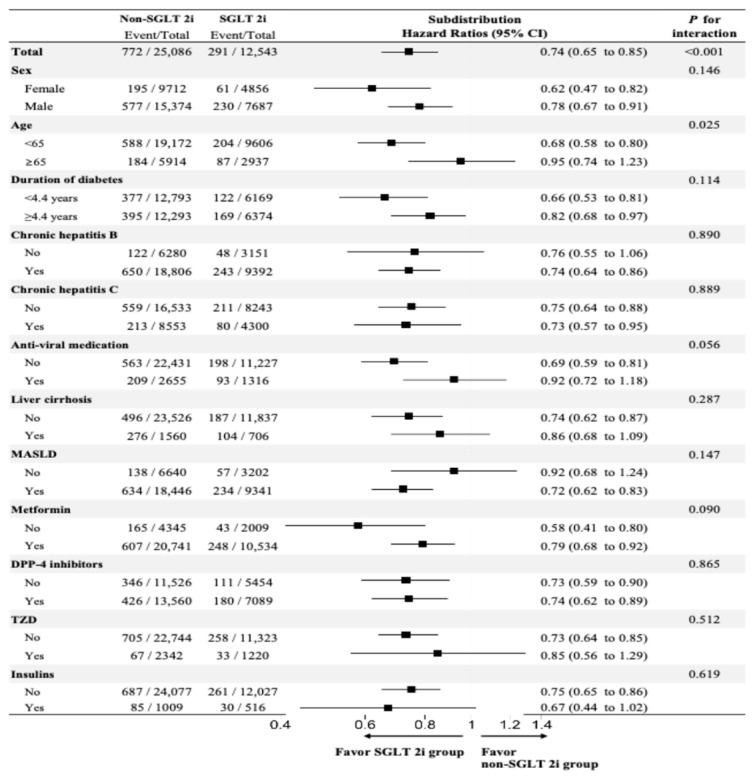
Subgroup analysis for composite liver-related complications. In the forest plot, the black squares represent the subdistribution hazard ratios (sHRs), and the horizontal lines represent the 95% confidence intervals (CIs). Bolded terms indicate the subgroup categories used for analysis.

Subgroup analyses were conducted by stratifying sex, age, diabetes duration, type of chronic viral hepatitis, antiviral agents, presence of cirrhosis or MASLD, and type of concurrent diabetes medication to confirm the different associations across subgroups (Figure 3). When we performed stratified analyses by age (≥65 years), diabetes duration (≥4.4 years), type of chronic viral hepatitis (CHB or CHC), presence of cirrhosis, and type of concurrent diabetes medication (metformin, DPP4i, TZD, or insulin), the decreased risk of liver-related events, including HCC, in the SGLT2i group was generally consistent regardless of the baseline characteristics with an sHR of 0.74 (95% CI, 0.65–0.85; *p* < 0.001). Notably, the risk reduction appeared more pronounced in patients younger than 65 years (interaction *p* = 0.025), suggesting a potential age-related difference in treatment effect.

## 4. Discussion

The role of metabolic dysfunction, including diabetes and obesity, is well established in the development of liver diseases, such as hepatic steatosis, fibrosis, and HCC, particularly in patients with chronic viral hepatitis [16]. Previous studies suggest that SGLT2is may have beneficial effects in patients with MASLD and metabolic dysfunction-associated steatohepatitis (MASH). For instance, a randomized controlled trial demonstrated significant improvement in liver fibrosis in patients treated with ipragliflozin compared to the control group [17]. Additionally, retrospective studies have highlighted the antifibrotic effects of SGLT2is, as indicated by improvements in the Fibrosis-4 index in patients with MASLD and T2DM [18].

Our study further corroborated these findings, showing that SGLT2i treatment lowers the risk of cirrhosis by 33% in patients with chronic viral hepatitis, irrespective of the presence of fatty liver disease. Moreover, SGLT2i use was significantly associated with a reduced risk of HCC, while no such protective effect was observed for other cancer types. This corroborates the findings of a recent cohort study from Hong Kong that reported a reduced risk of HCC in patients with T2DM and CHB who were treated with SGLT2is [19]. Collectively, these data suggest that SGLT2is significantly reduce composite liver-related complications, including HCC, by almost 36%.

The mechanisms underlying the hepatoprotective effects of SGLT2is are multifaceted and not yet fully understood. In a primary MASLD population, the efficacy of SGLT2is is well-documented to promote weight loss and improve insulin resistance, both of which are key factors in the progression of hepatic steatosis and fibrosis [20]. Notably, a recent meta-analysis of randomized clinical trials involving 1950 patients confirmed these benefits, demonstrating that SGLT2i treatment significantly reduces hepatic steatosis and improves liver function parameters [21]. In our cohort of patients with chronic viral hepatitis, we believe the hepatoprotective mechanism extends beyond simple metabolic correction. Chronic viral infection induces a state of persistent immune activation and oxidative stress. SGLT2is may exert anti-inflammatory effects by inhibiting inflammatory cytokines [22,23]. Specifically, SGLT2is suppress the activation of the nucleotide-binding domain, leucine-rich–containing family, pyrin domain–containing-3 (NLRP3) inflammasome—a key driver of inflammation—and inhibit fibrosis progression in MASLD and MASH [24]. This pathway is also implicated in HCC development, further highlighting the potential role of SGLT2is in reducing the risk of liver cancer [25,26]. In the context of viral hepatitis, this chronic inflammatory milieu is a major driver of compensatory proliferation and genomic instability, which ultimately leads to HCC [27]. By modulating these pathways, SGLT2is may provide a ‘dual hit’ protection: first, by mitigating the metabolic dysfunction that often coexists with and exacerbates viral liver disease, and second, by directly inhibiting the fibrogenic and oncogenic signaling pathways triggered by chronic viral infection [28].

Previous nationwide cohort studies comparing the effects of SGLT2is and other OADs, such as DPP4is and TZD, suggest the unique hepatoprotective benefits of SGLT2is [12,13]. Our study adds to this body of evidence by comparing patients who had never used SGLT2is with those who had used SGLT2is for at least 3 months. Several studies have reported the protective effects of metformin not only in preventing HCC but also in reducing the progression to liver-related death or transplantation in patients with chronic viral hepatitis [29,30,31]. Additionally, DPP4is are associated with insulin resistance and adipose tissue inflammation and are primarily recognized for their ability to improve liver-related outcomes in patients with MASLD [32]. Consequently, both metformin and DPP4is may have beneficial effects on liver outcomes in patients with chronic viral hepatitis, potentially preventing further deterioration and even improving the clinical course. Furthermore, after minimizing selection bias through PS matching between patients treated with SGLT2i and those treated with other OADs, our study demonstrated that the addition of SGLT2is resulted in superior liver-related outcomes than those of OADs alone. Although both metformin and DPP4is have been acknowledged for their positive effects on liver-related outcomes, our findings suggest a synergistic effect when combined with SGLT2is in patients with chronic viral hepatitis.

The enhanced efficacy of SGLT2is in patients under 65 years of age indicates a potential age-dependent heterogeneity in treatment response. This differential effect may be attributable to variations in hepatic metabolic reserve, disease progression trajectories, or differential pharmacodynamic responses across age strata.

The findings of this study must be interpreted cautiously due to its limitations. First, this nationwide cohort lacked specific laboratory data on HBV-DNA/HCV-RNA levels and direct fibrosis staging (e.g., FibroScan). However, we addressed this by adjusting for various clinical surrogates, including baseline liver enzymes, cirrhosis status, and the use of antiviral therapies. Additionally, key metabolic and lifestyle confounders—such as BMI, waist circumference, blood pressure, smoking, alcohol use, and physical activity—were rigorously balanced between the groups. While some residual confounding may persist due to the lack of viral load data, the inclusion of these comprehensive parameters ensures the robustness of our findings in representing the disease status of CHB/CHC patients. Second, most patients were included based on their medication prescriptions. Medication histories in electronic health records may not reflect actual drug exposure, and the duration of the SGLT2i treatment was ascertained based on these records. Furthermore, PS matching did not account for the number of antidiabetic drugs or glycemic control status, such as time-averaged or baseline glycated hemoglobin (HbA1c). Third, the relatively healthy study population in the Korean NHIS database with health examination data could have caused a selection or prescription bias. Moreover, we used a large dataset from a nationwide cohort sample. Thus, there may have been a selection bias. However, this study was designed with adequate PS matching to overcome this bias. Fourth, the median follow-up duration was just over three years, which may be insufficient to fully capture the long-term impact of SGLT2i on liver-related events. Longer follow-up studies with larger event numbers are needed to confirm these findings. Fifth, while FLI can be influenced by hepatic inflammation, its clinical validity as a prognostic marker for HCC and mortality has been well-documented in various chronic viral hepatitis cohorts, and we further minimized potential confounding by balancing baseline liver function through propensity score matching [33,34,35]. Finally, cirrhosis and liver-related complications in this study were identified through diagnostic and prescription codes rather than direct diagnostic measures, such as imaging or elastography. Hence, there may be some limitations in the precise identification of liver-related complications, including cirrhosis, compared to other endpoints, such as malignancy or mortality. However, the definitions employed in previous nationwide cohort studies have been proven as reliable and reproducible.

Despite these limitations, to the best of our knowledge, this is the first large-scale study to investigate the effect of SGLT2i treatment in patients with chronic viral hepatitis.

## 5. Conclusions

Our study suggests that SGLT2is offer significant protection against composite liver-related complications, including cirrhosis and HCC, in patients with T2DM and chronic viral hepatitis. These benefits are likely mediated by improvements in metabolic function, a reduction in inflammation, and the inhibition of fibrosis progression. Although further research is needed to elucidate the exact mechanisms involved and explore the long-term impact of SGLT2is, this discovery raises the possibility that SGLT2is could serve as a therapeutic option for preventing HCC and cirrhosis in patients with CHB/CHC and metabolic disorders, especially those with long-standing diabetes.

## Figures and Tables

**Table 1:** Baseline characteristics after propensity score matching.

	Non-SGLT2i Group *n* = 25,086	SGLT2i Group ** n = 12,543	ASMD
Age, mean (SD)	58.2 (9.0)	58.1 (9.2)	0.016
Male, *n* (%)	15,374 (61.3)	7687 (61.3)	0
Time to index date from entry of cohort (years), median (IQR) *	1.9 (0.5–4.8)	2.0 (0.3–4.9)	0.009
Time to index date from T2DM diagnosis (years), median (IQR)	7.0 (3.1–10.4)	7.3 (3.2–10.4)	0.025
Time to index date from CHB or CHC (years), median (IQR)	4.3 (1.5–8.6)	4.3 (1.7–8.5)	0.019
Chronic hepatitis B ^¶^	18,806 (75.0)	9392 (74.9)	0.002
Chronic hepatitis C ^¶^	8553 (34.1)	4300 (34.3)	0.004
Social economic status			<0.001
Lower	6596 (26.3)	3286 (26.2)	
Middle	7416 (29.6)	3706 (29.5)	
High	11,074 (44.1)	5551 (44.3)	
Comorbidity ^†^, *n* (%)			
Fatty liver index, mean (SD)	49.7 (25.6)	51.2 (25.9)	0.056
MASLD			
FLI ≥ 30	18,446 (73.5)	9341 (74.5)	0.021
FLI ≥ 60	9343 (37.2)	4981 (39.7)	0.051
Liver cirrhosis	1560 (6.2)	706 (5.6)	0.025
Hypertension	14,848 (59.2)	7550 (60.2)	0.020
Dyslipidemia	17,978 (71.7)	8946 (71.3)	0.008
Cardiovascular disease	5650 (22.5)	2842 (22.7)	0.003
Concurrent drug treatment ^†^, *n* (%)			
Anti-viral medication for CHB or CHC	2655 (10.6)	1316 (10.5)	0.003
Anti-diabetic agents			
Metformin	20,741 (82.7)	10,534 (84.0)	0.035
DPP-4 inhibitor	13,560 (54.1)	7089 (56.5)	0.050
Sulfonylurea	9299 (37.1)	4761 (38.0)	0.018
TZD	2342 (9.3)	1220 (9.7)	0.013
GLP1 agonist	108 (0.4)	82 (0.7)	0.030
Insulins	1009 (4.0)	516 (4.1)	0.005
Antihypertensives			
RAS inhibitor	8362 (33.3)	4312 (34.4)	0.022
Calcium channel blocker	8910 (35.5)	4589 (36.6)	0.022
β blocker	3197 (12.7)	1738 (13.9)	0.033
Diuretics	4241 (16.9)	2281 (18.2)	0.034
Lipid-lowering agents			
Statins	16,469 (65.7)	8267 (65.9)	0.006
Others	7103 (28.3)	3516 (28.0)	0.006
Antiplatelet agents	7703 (30.7)	3961 (31.6)	0.019
Anticoagulant agents	693 (2.8)	341 (2.7)	0.003
Smoking, *n* (%)			0.029
never	13,244 (52.8)	6568 (52.4)	
former	8037 (32.0)	4002 (31.9)	
current	3805 (15.2)	1973 (15.7)	
Alcohol consumption, *n* (%)			<0.001
never	12,332 (49.2)	6201 (49.4)	
≤2 times/week	6355 (25.3)	3149 (25.1)	
≥3 times/week	6399 (25.5)	3193 (25.5)	
Physical activity, *n* (%)			0.029
never	10,227 (40.8)	5184 (41.3)	
≤2 times/week	4335 (17.3)	2064 (16.5)	
≥3 times/week	10,524 (42.0)	5295 (42.2)	
BMI, mean (SD)	26.3 (3.3)	26.7 (3.4)	0.106
Waist circumference, mean (SD)	88.1 (8.7)	88.8 (8.9)	0.082
Total cholesterol, (mg/dL), mean (SD)	183.1 (44.2)	181.3 (45.9)	0.040
LDL-C (mg/dL), mean (SD)	102.0 (41.1)	100.2 (40.3)	0.042
HDL-C (mg/dL), mean (SD)	50.1 (12.9)	49.8 (12.7)	0.020
TG-C (mg/dL), median (IQR)	134 (94–194)	132 (94–191)	0.015
SBP, mean (SD)	128.0 (14.4)	128.3 (14.8)	0.015
DBP, mean (SD)	78.6 (9.8)	78.7 (10.0)	0.009
FBS (mg/dL), mean (SD)	145.6 (49.0)	144.8 (44.4)	0.018
Creatinine (mg/dL), mean (SD)	0.88 (0.26)	0.87 (0.24)	0.017
AST, mean (SD)	39.0 (40.6)	38.8 (30.5)	0.006
ALT, mean (SD)	43.9 (53.5)	43.8 (36.9)	0.002
γ-GTP, mean (SD)	66.4 (87.8)	65.0 (85.3)	0.016

Abbreviations: CHB, chronic hepatitis B virus; CHC, chronic hepatitis C virus; T2DM, type 2 diabetes mellitus; SGLT2i, sodium-glucose co-transporter 2 inhibitors; ICD-10, International Classification of Disease, Tenth Revision; TZD, thiazolidinedione; DPP4i, dipeptidyl peptidase-4 inhibitors; ASMD, absolute standardized mean difference; IQR, interquartile range; GLP1, Glucagon-Like Peptide 1; RAS, renin-angiotensin system; SD, standard deviation; PS, propensity score; BMI, body mass index; LDL-C, low-density lipoprotein cholesterol; HDL-C, high-density lipoprotein cholesterol; TG-C, triglycerides cholesterol; SBP, systolic blood pressure; DBP, diastolic blood pressure; FBS, fasting blood sugar; AST, aspartate aminotransferase; ALT, alanine aminotransaminase; γ-GTP, γ-glutamyl transpeptidase. * Cohort entry is defined as the later date between the diagnosis of DM and the diagnosis of CHB/CHC. ** The index date is defined as the initiation date of SGLT-2i prescription, and for the control group, it is set as 1 July of the same year as the matched SGLT-2i group’s prescription initiation. ^†^ Comorbidity and concurrent drug treatment are defined as the presence of diagnosis codes, or the use of medications identified within 1 year prior to the index date. ^¶^ Patients with concurrent chronic hepatitis B and chronic hepatitis C were included: 9.2% in the SGLT2i group (*n* = 1149) and 9.1% in the non-SGLT2i group (*n* = 2273), ASMD < 0.001.

**Table 2:** Risk of incident HCC or liver-related complication in SGLT2i and non-SGLT2i groups.

	No. of Events (IR per 1000 PY)	Subdistribution Hazard Ratio * (95% CI)	*p*-Value
*Composite liver-related complications*			
Non-SGLT 2i group (*n* = 25,086)	772 (8.99)	1 (Reference)	
SGLT 2i group (*n* = 12,543)	291 (6.67)	0.74 (0.65–0.85)	<0.001
HCC			
Non-SGLT 2i group	515 (5.96)	1 (Reference)	
SGLT 2i group	202 (4.61)	0.77 (0.66–0.91)	0.002
Cirrhosis-related complication			
Non-SGLT 2i group	358 (4.13)	1 (Reference)	
SGLT 2i group	115 (2.61)	0.64 (0.52–0.79)	<0.001
Liver transplant			
Non-SGLT 2i group	60 (0.69)	1 (Reference)	
SGLT 2i group	13 (0.29)	0.44 (0.24–0.81)	0.008
Liver-related mortality			
Non-SGLT 2i group	197 (2.23)	1 (Reference)	
SGLT 2i group	57 (1.29)	0.67 (0.50–0.91)	0.010

Abbreviations: SGLT2i, sodium-glucose co-transporter 2 inhibitors; IR, incidence rate; PY, person-years; HCC, hepatocellular carcinoma; CI, confidence intervals. * Non-liver related death or LT were considered as competing events.

**Table 3:** Risk of mortality and the development of new-onset liver cirrhosis.

	No. of Events (IR per 1000 PY)	Hazard Ratio (95% CI)	*p*-Value
*New-onset liver cirrhosis* *in patients without previous LC history*			
Non-SGLT 2i group (*n* = 23,526)	320 (3.96)	1 (Reference)	
SGLT 2i group (*n* = 11,837)	110 (2.66)	0.67 (0.54–0.83) *	<0.001
*All-cause mortality*			
Non-SGLT 2i group (*n* = 25,086)	695 (7.88)	1 (Reference)	
SGLT 2i group (*n* = 12,543)	256 (5.79)	0.76 (0.65–0.88)	<0.001

Abbreviations: SGLT2i, sodium-glucose co-transporter 2 inhibitors; IR, incidence rate per 1000 PY; CI, confidence intervals. * All-cause death was considered as competing events.

## Data Availability

The data that support the findings of this study are available from public datasets, with restrictions.

## Supplementary Materials

The following supporting information can be downloaded at: https://www.mdpi.com/article/10.3390/cancers18010120/s1, Figure S1: Scheme of study design; Table S1: Baseline characteristics before PS matching; Table S2: Risk of Incident Malignancy, Except HCC, in the Two Groups.

## Abbreviations

ASMD, absolute standardized mean difference; CHB, chronic hepatitis B; CHC, chronic hepatitis C; DPP4i, dipeptidyl peptidase-4 inhibitors; HR, hazard ratio; ICD-10, International Classification of Disease, Tenth Revision; MASLD, metabolic dysfunction-associated steatotic liver disease; MASH, metabolic dysfunction-associated steatohepatitis; NHIS, National Health Insurance Service; NLRP3, nucleotide-binding domain, leucine-rich–containing family, pyrin domain-containing 3; OADs, oral antidiabetic drugs; PS, propensity score; SGLT2i, sodium-glucose cotransporter-2 inhibitors; T2DM, Type 2 diabetes mellitus; TZD, thiazolidinedione.
