# Supplementary material for: Association Between SGLT2 Inhibitor Use and Reduced Risk of Liver-Related Events, Including Hepatocellular Carcinoma, in Diabetic Patients with Viral Hepatitis: A Nationwide Cohort Study
Source: Cancers (Basel). 2025 Dec 30;18(1):120. doi: 10.3390/cancers18010120 (PMC12784983; doi:10.3390/cancers18010120)
Supplement: Supplementary file 1 [file cancers-18-00120-s001.zip › cancers-3995176-supplementary.pdf]

Table S1. Baseline characteristics before PS matching.

	Non-SGLT2i group n=59,502	SGLT2i group n=15,588	ASMD
Age, mean (SD)	61.6 (10.5)	55.8 (10.2)	0.562
Male, n (%)	34989 (58.8)	9808 (62.9)	0.084
Time to index date from entry of cohort (years), median (IQR)	1.4 (0.2–3.9)	2.0 (0.3–4.9)	0.149
Time to index date from DM diagnosis (years), median (IQR)	6.6 (3.1–10.1)	7.3 (3.2–10.4)	0.063
Time to index date from CHB or CHC (years), median (IQR)	3.4 (0.9–7.5)	4.4 (1.7–8.6)	0.209
Chronic hepatitis B	43986 (73.9)	11744 (75.3)	0.033
Chronic hepatitis C	20831 (35.0)	5273 (33.8)	0.025
Concurrent CHB and CHC	5315 (8.9%)	1429 (9.2%)	0.023
Social economic status			0.024
Lower	15585 (26.2)	4001 (25.7)	
Middle	17122 (28.8)	4711 (30.2)	
High	26795 (45.0)	6876 (44.1)	
Comorbidity †, n (%)			
Fatty liver index, mean (SD)	43.5 (26.1)	55.9 (26.8)	0.467
MASLD	37507 (63.0)	12257 (78.6)	0.348
Liver cirrhosis	3905 (6.6)	840 (5.4)	0.049
Hypertension	34837 (58.5)	9440 (60.6)	0.041
Dyslipidemia	36479 (61.3)	11398 (73.1)	0.254
Cardiovascular disease	12384 (20.8)	3590 (23.0)	0.054
Concurrent drug treatment†, n (%)			
Antiviral medication for chronic hepatitis B/C	6269 (10.5)	1632 (10.5)	0.002
Anti-diabetic agents			
Metformin	47080 (79.1)	13065 (83.8)	0.121
DPP-4 inhibitor	32798 (55.1)	8510 (54.6)	0.011
Sulfonylurea	18018 (30.3)	6200 (39.8)	0.200
TZD	4642 (7.8)	1554 (10.0)	0.076
GLP1 agonist	247 (0.4)	104 (0.7)	0.034
Insulins	2000 (3.4)	702 (4.5)	0.059
Antihypertensives			
RAS inhibitor	19187 (32.2)	5396 (34.6)	0.050
Calcium channel blocker	21584 (36.3)	5694 (36.5)	0.005
β blocker	7534 (12.7)	2256 (14.5)	0.053
Diuretics	10887 (18.3)	2810 (18.0)	0.007
Lipid-lowering agents			
Statins	34456 (57.9)	10307 (66.1)	0.170
Others	12404 (20.8)	4693 (30.1)	0.214
Antiplatelet agents	18785 (31.6)	4688 (30.1)	0.032
Anticoagulant agents	1347 (2.3)	498 (3.2)	0.057
Smoking, n (%)			0.109
never	33218 (55.8)	7969 (51.1)	
former	17803 (29.9)	4975 (31.9)	
current	8481 (14.3)	2644 (17.0)	
Alcohol consumption, n (%)			0.133
never	32263 (54.2)	7444 (47.8)	
≤2 times/week	12939 (21.7)	4216 (27.0)	
≥3 times/week	14300 (24.0)	3928 (25.2)	
Physical activity, n (%)			0.082
never	25420 (42.7)	6392 (41.0)	
≤2 times/week	8805 (14.8)	2808 (18.0)	
≥3 times/week	25277 (42.5)	6388 (41.0)	
BMI, mean (SD)	25.5 (3.5)	27.5 (4.0)	0.528
Waist circumference, mean (SD)	86.5 (8.9)	90.5 (9.9)	0.424
Total cholesterol, (mg/dL), mean (SD)	180.5 (43.6)	183.7 (46.1)	0.070
LDL-C (mg/dL), mean (SD)	100.6 (39.6)	102.3 (43.8)	0.040
HDL-C (mg/dL), mean (SD)	50.7 (13.3)	49.2 (12.5)	0.115
TG-C (mg/dL), mean (SD)	124 (88–180)	138 (97–200)	0.193

SBP, mean (SD)	128.2 (14.8)	128.6 (14.8)	0.027
DBP, mean (SD)	77.8 (9.9)	79.4 (10.3)	0.159
FBS (mg/dL), mean (SD)	136.3 (42.7)	150.5 (51.4)	0.300
Creatinine (mg/dL), mean (SD)	0.88 (0.30)	0.88 (0.25)	0.034
AST, mean (SD)	37.2 (35.7)	40.6 (32.8)	0.098
ALT, mean (SD)	39.4 (47.9)	48.0 (44.9)	0.184
γ-GTP, mean (SD)	62.6 (91.2)	67.6 (114.9)	0.048

Abbreviations: CHB, chronic hepatitis B virus; CHC, chronic hepatitis C virus; T2DM, type 2 diabetes mellitus; MASLD, metabolic dysfunction-associated steatotic liver disease; SGLT2i, sodium-glucose co-transporter 2 inhibitors; ICD-10, International Classification of Disease, Tenth Revision; TZD, thiazolidinedione; DPP4i, dipeptidyl peptidase-4 inhibitors; ASMD, absolute standardized mean difference; IQR, interquartile range; GLP1, Glucagon-Like Peptide 1; RAS, renin-angiotensin system; SD, standard deviation; PS, propensity score; BMI, body mass index; LDL-C, low-density lipoprotein cholesterol; HDL-C, high-density lipoprotein cholesterol; TG-C, triglycerides cholesterol; SBP, systolic blood pressure; DBP, diastolic blood pressure; FBS, fasting blood sugar; AST, aspartate aminotransferase; ALT, alanine aminotransaminase; γ-GTP, γ-glutamyl transpeptidase.

Table S2. Risk of Incident Malignancy, Except HCC, in the Two Groups.

	**No. of event (IR per 1000 PY)**	**Subdistribution Hazard ratio* (95% CI)**	***P*-value**
Cholangiocarcinoma			
Non-SGLT 2i group (N=25086)	57 (0.65)	1 (Reference)	
SGLT 2i group (N=12543)	24 (0.54)	0.81 (0.49-1.32)	0.400
Pancreatic cancer			
Non-SGLT 2i group	85 (0.97)	1 (Reference)	
SGLT 2i group	37 (0.84)	0.81 (0.54-1.23)	0.322
Gallbladder cancer			
Non-SGLT 2i group	5 (0.06)	1 (Reference)	
SGLT 2i group	5 (0.11)	1.92 (0.53-7.00)	0.321
Colon cancer			
Non-SGLT 2i group	170 (1.95)	1 (Reference)	
SGLT 2i group	79 (1.79)	0.89 (0.67-1.17)	0.396
Gastric cancer			
Non-SGLT 2i group	148 (1.70)	1 (Reference)	
SGLT 2i group	64 (1.45)	0.84 (0.63-1.14)	0.268
Ampullar of Vater cancer			
Non-SGLT 2i group	10 (0.11)	–	
SGLT 2i group	0 (0)	–	
Bile duct cancer			
Non-SGLT 2i group	22 (0.25)	1 (Reference)	
SGLT 2i group	10 (0.23)	0.91 (0.43-1.90)	0.798

Abbreviations: SGLT2i, sodium-glucose co-transporter 2 inhibitors; IR, incidence rate; PY, person years; CI, confidence intervals.*All-cause death was considered as competing events.

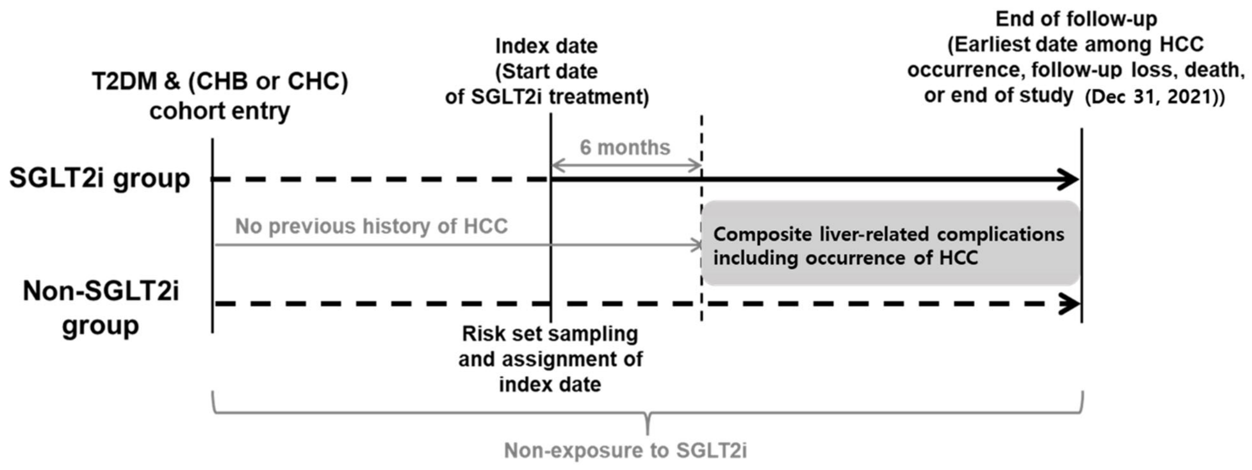

Figure S1. Scheme of study design.
